# Cancer Risk Assessment for Workers Exposed to Pollution Source, a Petrochemical Company, Iran

**DOI:** 10.18502/ijph.v49i7.3587

**Published:** 2020-07

**Authors:** Bahram HARATI, Seyed Jamaleddin SHAHTAHERI, Hossein Ali YOUSEFI, Ali HARATI, Ali ASKARI, Nabi ABDOLMOHAMADI

**Affiliations:** 1. Department of Occupational Health Engineering, School of Public Health, Tehran University of Medical Sciences, Tehran, Iran; 2. Department of Environmental Chemical Pollutants and Pesticides, Institute for Environmental Research, Tehran University of Medical Sciences, Tehran, Iran; 3. Department of Parasitology and Mycology, School of Medicine, Isfahan University of Medical Sciences, Isfahan, Iran; 4. Department of Occupational Health Engineering, School of Public Health, Boroujerd Branch, Islamic Azad University, Boroujerd, Iran; 5. Department of Chemical Engineering, School of Chemistry, Tehran University, Tehran, Iran

**Keywords:** Risk assessment, Cancer risk analysis, Volatile organic compounds, Hydrogen sulfide, Occupational, Petrochemical industry

## Abstract

**Background:**

Air pollution have led to severe problem of adverse health effect in the world. This study aimed to conduct the health risk assessment, cancer risk analysis, and non-cancer risk for exposure to volatile organic compounds (VOCs) and hydrogen sulfide (H _2_ S) in petrochemical industry.

**Methods:**

In this cross-sectional research, 123 samples were collected in the ambient air in Iran during winter 2016. For sampling and analysis of VOCs and H _2_ S, 3 methods (numbers 1500, 1501, and 6013) presented by the National Institute of Occupational Safety and Health (NIOSH) were used. For determination of risk assessment of chemical pollutants, semi-quantitative method presented by the Occupational Safety and Health Division, Singapore was used. Finally, for calculation of cancer risk analysis, Chronic Daily Intake (CDI) and calculation of non-cancer risk, Exposure Concentration (EC) were used.

**Results:**

Average concentration of benzene (2.12±0.95) in breathing zone of workers were higher than the Threshold Limit Values-Time Weighted Average (TLV-TWA) (*P*<0.05). Among chemical substance, benzene had very high rank of risk in petrochemical industry. Rank of risk for H _2_ S, toluene, and xylene present in the breathing zone of workers was low. The mean cancer risk for workers exposed to benzene was estimated 8.78×10^−3,^ in other words, 8.7 cancer per 1000 i.e. higher than the acceptable standard of 10^−6^. In our study, non-cancer risk for BTX was higher than the acceptable standard of 1.

**Conclusion:**

In particular, overall cancer and toxic risk can be associated with long term exposure to benzene.

## Introduction

During the last years, technology in major industries has grown, despite the advance stage can cause emission largest pollutions in the workplace that we still unknown environment hazards (1).

Emission of different levels concentration of chemical substances into the atmosphere may play main role in the ozone, photochemical oxidant, and greenhouse effects (2, 3). Long term exposure to pollutants in the ambient air can result in adverse health effects (4, 5). Four million people worldwide are employed in the chemical industries (6).

One of the most important pollutants released in the industrial processes is hydrogen sulfide (H _2_ S). Smell of this gas is rotten eggs with colorless and high toxic effects (7). Initial reports about toxicity of H _2_ S were published in 1713, so that, next investigations for consideration on the H _2_ S were assigned for checking toxic effects (7). Some studies showed, systemic (8) and physiological effects (9) of H _2_ S.

Volatile organic compounds (VOCs) are some of chemical substances generated evaporative releases from different fossil fuels processing steps (10, 11). VOCs released from chemical laboratories can cause high cancer risk among workers (12). 37 VOCs were detected in the ambient air of university in Hong Kong (13). VOCs including benzene, toluene, and xylene (BTX) has adverse health effects, such as hematopoietic (14, 15) neurological effects on (CNS) systems (16, 17). Benzene is classified in class 1 human and animals carcinogenic recommended by International Agency of Research on Cancer (IRAC) (1982) (18). Among the 23 VOCs detected in the breathing zone of workers, benzene had the highest concentration (19). Long term exposure to benzene released from oil process may result in the occurrence of leukemia (18, 20, 21). Health effect of xylene released from oil process including CNS, irritation of eye and throat impairment (22). Among BTX compounds, cancer risk of benzene in the gas station was higher than the standard level recommended by EPA guideline (23).

Risk assessment and cancers risk analysis of chemical substances are essential for management programs enacting appropriate for reduced exposure workers (24). The risk assessment can be considered different fields for identifying, assessing, and planning for potential harmful health effects on the workers exposed to the chemicals (25). The high rate of concern to cancer risk have been reported in petrochemicals, oil, and gas industrials (26, 27). Risk assessment for exposure to various levels of chemical substances in the ambient air has been conducted by some studies (28, 29). Risk assessment including 3 steps such as problem formulation, planning, and risk management (30). Health risk assessment at the right time can help us to provide information about concentrations of chemical substances in the workplace, prioritize the ranking of hazard, and increased predict efficiency (31).

This study aimed to conduct the health risk assessment of VOCs and H _2_ S as well as cancer risk analysis and non-cancer risk of as benzene, toluene, and xylene (BTX) at a petrochemical industry.

## Materials and Methods

This investigation was cross-sectional research for assessment of rank of pollutants risk releases at a petrochemical industry in Iran. This study was conducted during winter 2016. Overall, 123 samples (50 samples for workers exposed to VOCs, 70 samples for workers exposed to H _2_ S, and 3 samples for blank (control)) were collected in the ambient air of petrochemical industry. Consent form was completed for all participants before they participated in the research. Inclusion criteria in the present study were exposed to more than 4 h a day with pollution.

### Sampling and analysis of VOCs

Sampling and analysis of VOCs were performed using of 2 methods (numbers of 1500 and 1501) presented by the National Institute of Occupational Safety and Health (NIOSH). Coconut shell charcoal (100/50 mg) was used for collecting air sampling of VOCs in the breathing zone of workers. Before personal sampling, micropump was calibrated in the flow rate of 0.01 L/min by representative sampler in line. After collection, CS _2_ (1 ml) were used for extraction of analyte. Gas Chromatography-Flame Ionization Detector (GC-FID) VARIAN c-3800 was used for analysis of chemical compounds.

### Sampling and analysis of H _2_ S

Sampling and analysis of H _2_ S were performed using method number of 6013 presented by the NIOSH. Coconut shell charcoal (400/200 mg) was used for air sampling of H _2_ S in the breathing zone of workers. Before air sampling, personal sampling pump was calibrated in the flow rate of 2 L/min by representative sampler in line. After collection, NH _4_ OH (2 ml of 0.2 M) and 5 ml H_2_O _2_ were used for extraction of analyte. Chemical analyses were performed by Ion Chromatography.

After analysis of VOCs and H _2_ S, the next step was to determine concentration of pollutants in the breathing zone of workers.

Calculate concentration of analyte in the air volume was defined by Eq.1.

$$C=(W_{f}+W_{b}-B_{f}-B_{b})/V$$

W _f_ : analyte found in the sample front (Coconut shell charcoal)
W _b_ : analyte found in the sample back (Coconut shell charcoal)
B _f_ : average media in the blank front (Coconut shell charcoal)
B _b_ : average media in the blank back (Coconut shell charcoal)
V: air volume sample (L)
C: concentration of pollutant (mg/m ^3^)

Concentration of pollutant in Eq.1 come in form mg/m ^3^ , calculation mg/m ^3^ to parts per million (ppm) in the vapor pressure 760 mmHg, using the form of Eq.2.

$$\mathrm{PPM}=\mathrm{mg}/m^{3}\times 24.45/M$$

M: molecular weight (benzene=78.11)

Overall, 120 air samples were collected from 60 workers (two samples from each workers). Duration time for taking all samples was 360 h (3 horse per sample). For calculation of time-weight average (TWA) using the form of Eq.3.

$$\mathrm{TWA}=C_{1}T_{1}+C_{2}T_{2}/8$$

C: concentration of pollutant (ppm)
T: duration time of sampling (hour)

### Risk assessment method

For determination of risk assessment of chemical pollutants in the workers breathing zone, semi-quantitative method presented by the Occupational Safety and Health Division, 18 Havelock Road, Ministry of Manpower, Singapore was used (32).

### Stage 1: Hazard Rating (HR)

After identification of chemical pollutants in the workplace, the next step was to determine toxic or harmful effects of chemical. HR can be determined from toxic or harmful effects (Table 1).

**Table 1:** Hazard Rating (HR)

*** Gases ***	*** Hazard Rating ***	*** Description of effects/Hazard category ***
Benzene	5	-IARC group 1 -ACGIH A1 carcinogens
Toluene, Xylene, Hydrogen sulfide (H _ 2 _ S )	3	-IARC group 2B -ACGIH A3 carcinogens
Pentane, Hexane, Heptane, Octane, Nonae	1	-No known adverse health effects -ACGIH A5 carcinogens

### Stage 2: Exposure Rating (ER)

Exposure rating (ER) can be determined, using actual exposure level. Weekly exposure (ppm or mg/m ^3^) was calculated by Eq.4.

$$\text{E}=\frac{\text{F}\times \text{D}\times \text{M}}{\text{W}}$$

E: weekly exposure (ppm or mg/m ^3^)
F: frequency of exposure per week (no. per week)
D: average duration of each exposure (hours)
M: magnitude of exposure (ppm or mg/m ^3^)
W: average working hours per week (40 h)

ER assessment can be determined from compared weekly exposure (E) than to the PEL (Long Term) (Table 2).

**Table 2:** Exposure Rating (ER)

*** E/PEL ***	*** Exposure Rating (ER) ***
<0.1	1
0.1 to <0.5	2
0.5 to <1.0	3
1.0 to <2.0	4
≥2.0	5

PEL: Corresponding permissible exposure level

### Stage 3: calculation of Risk Level

Risk levels were using Eq.5.

Risk Level=√HR×FR

HR: Hazard rating on the scale of 1 to 5 (see Table 1)
ER: Exposure rating on the scale of 1 to 5 (see Table 2)

### Stage 4: Significance of risk

Rank of each risk was determined with following Table 3.

**Table 3:** Risk rating

*** Risk rating ***	*** Ranking ***
1	Negligible
2	Low
3	Medium
4	High
5	Very High

### Cancer and non-cancer risk calculations

The method of cancer risk assessment was focused on assessing carcinogenic substances in the workplace. Long term exposure to benzene releases from chemical industrial may result in the occurrence of leukemia in workers (33). On the other hand, benzene can cause cancer even at a low-level of concentrations (27). Therefore, cancer risk analysis is essential for identification of hazardous substance and prioritize the ranking of hazard in the workplace. Cancer risk assessment Chronic Daily Intake (CDI) was defined by Eq.6.

$$\begin{array}{l} \mathrm{Cancer}\;\mathrm{risk}=\mathrm{CDI}\times \mathrm{CS}F_{i}\\ \;\;CDI=(CA\times IR\times ET\times EF\times ED)/(BW\times AT) \end{array}$$

CDI (mg/kg/day): Chronic Daily Intake
CA (mg/m ^3^): Contaminant Concentration in Air
IR (m ^3^ /h): Inhalation Rate (0.875 m ^3^ /h assumed for adult)
ET (h/day): Exposure Time (8 h/day for workers)
EF (day/years): Exposure Frequency (350 day/years assumed for workers)
ED (years): Exposure Duration (30 years for workers)
BW (kg): Body weight (60.54 kg, average body weight of workers)
AT (day): Averaging Time (70 years× 365 for cancer or ED × 365 for non-cancer)
CSFi (mg/kg/day) ^−1^ : inhalation cancer slope factor Cancer risk higher than 10 ^−6^ was considered carcinogenic effects of concern and a value ≤10 ^−6^ was considered an acceptable level.

Exposure Concentration (EC) for non-cancer risk:

Hazard Quotient (HQ) parameters for risk assessment of non-cancer condition is assessed as in Eq.7.
  $$\begin{array}{l} \text{HQ}=\text{EC}/\text{Rfc}\\ \text{EC}=(\text{CA}\times \text{ET}\times \text{EF}\times \text{ED})/\text{AT} \end{array}$$
Rfc (μg/m ^3^ or ppb): Represent exposure concentration

HQ >1 mean adverse non-carcinogenic effects of concern, a value HQ of ≤1 was considered acceptable level.

### Statistics analysis

For analysis of data, SPSS ver. 23 (Chicago, IL, USA), was used. Comparison study performed between the mean concentration of pollutants (benzene, toluene, xylene, pentane, hexane, heptane, octane, nonae and H2S) in breathing zone of workers with standard threshold limit value (TLV) was by using t-test. A *P*-value<0.05 was considered for significances evaluation.

## Results

### Personal air VOCs

Fifty samples of VOCs were collected from the workplace. The average benzene, toluene, xylene, pentane, hexane, heptane, octane, and nonae exposure levels in exposed subjects were 2.12±0.95, 9.84±2.53, 11.87±4.44, 0.13±0.05, 0.16±0.05, 6.45±2.44, 0.15±0.05, and 0.14±0.55 ppm respectively (Table 4). Average concentration of benzene (2.12±0.95) in breathing zone of workers were higher than the Threshold Limit Values-Time Weighted Average (TLV-TWA) (*P*<0.05) recommended by the American Conference of Governmental Industrial Hygienists (ACGIH). Average concentrations of toluene, xylene, pentane, hexane, heptane, octane, and nonae were significantly lower than the TLV-TWA recommended by the ACGIH for all gases (*P*<0.05).

**Table 4:** Exposure levels of volatile organic compounds by categories, in workers of petrochemical industry

*** Concentration ***	*** TWA (mean ± SD) ***	*** Range ***	*** TLV-TWA (AC- GIH) (ppm) ***	*** P-value ***
Benzene	2.12±0.95	0.2–9.2	0.05	0.041
Toluene	9.84±2.53	0.09–17.10	50	0.001
Xylene	11.87±4.44	7.3–32.80	100	0.001
Pentane	0.13±0.05	0.03–0.22	600	0.001
Hexane	0.16±0.05	0.01–0.28	50	0.001
Heptane	6.45±2.44	0.07–10.2	400	0.001
Octane	0.15±0.05	0.03–0.28	300	0.001
Nonae	0.14±0.55	0.03–0.22	200	0.001

### Personal air H _2_ S

Seventy samples of H _2_ S were collected in the workplace. The TLV-TWA H _2_ S exposure content in air is 10 ppm. The mean H _2_ S level and standard deviation of the exposed-pollutant was 0.22±0.45 ppm (Table 5). The concentration of H _2_ S in the ambient air was lower than the TLVTWA recommended by ACGIH (*P*<0.05).

**Table 5:** Exposure levels of hydrogen sulfide (H _2_ S) by categories, in workers of petrochemical industry

*** Concentration ***	*** TWA (mean ± SD) ***	*** Range ***	*** P-value ***
hydrogen sulfide (H _ 2 _ S )	0.22±0.45	0.02–3.0	0.001

### Risk assessment

Table 3 presents the risk assessment and ranking of pollutants for exposure to VOCs and H _2_ S on workers of petrochemical industry. Detected benzene among chemical substances had very high rank of risk in petrochemical industry. Rank of risk for H _2_ S, toluene, and xylene in the breathing zone of workers was low (L). In other cases, risk ranks were in negligible rate (N) (Table 6).

**Table 6:** The results of risk assessment based on various concentrations of gases

** Gases **	** Hazard Rating **	** Exposure Rating **	** Risk Rating **	** Ranking **
Benzene	5	5	5	Very High
Toluene	3	1	1.73	Low
Xylene	3	2	2.44	Low
Pentane	1	1	1	Negligible
Hexane	1	1	1	Negligible
Heptane	1	1	1	Negligible
Octane	1	1	1	Negligible
Nonae	1	1	1	Negligible
Hydrogen sulfide (H _ 2 _ S )	3	1	1.73	Low

### Cancer risk and non-cancer assessment

For calculation of cancer risk, Chronic Daily In-take (CDI) and for calculated of non-cancer risk, Exposure Concentration (EC) was used. The mean cancer risk for workers exposed to benzene was estimated 8.78×10 ^−3^ (Table 4). The CDIs for benzene was 0.321 (mg/kg/day). The ECs for benzene, toluene, and xylene were 22.25, 103.35 and 123.72 (mg/m ^3^), respectively. The cancer risks of benzene was higher than the acceptable limit of 10 ^−6^ . The non-cancer risks for benzene, toluene, and xylene were 741.66, 21.64 and 156.60, respectively (Table 7).

**Table 7:** The average lifetime cancer risk and non-cancer assessments for BTX compounds

** BTX compounds **	** EC (mg/m *^ 3 ^* ) **	** Non-cancer risk (HQ) **	** CSFi (mg/kg/day) *^ −1 ^***	** CDI (mg/kg/day) **	** Cancer risk **
	Benzene	22.25	741.66	2.73×10 ^ −2 ^	0.321	8.78×10 ^ −3 ^
	Toluene	103.35	21.64	-	-	-
	Xylene	123.72	156.60	-	-	-

## Discussion

Risk assessment uses qualitative or quantitative techniques provided ranking of chemical dangerous (18, 34). Long term exposure to various levels concentration of pollutants may cause increased risk of cancer (35). Using of fossil fuels (coal, gas, and oil) in the various industries (36) can result in emission of several substances into the atmosphere producing greenhouse effects (37).

This study indicated that concentration of pentane in the breathing zone of workers was lower than the other concentration of VOCs. Average concentration of xylene was higher than the other cases. Average concentration of benzene was higher than the TLV-TWA recommended by ACGIH. While other concentrations of pollutants were lower than the standard levels. Vapor pressure of VOCs can be considered as the main reason for distribution of substances in the ambient air (38). In Taiwan petroleum, daily maximum concentration of benzene was 82 ppb (39). IRAC statistic evaluations indicate that 400000 to 500000 persons in the world have been employed in the petroleum (40).

In the last two decades, high mechanization and automatization of petroleum industries have resulted in reduction of workforce (40). In our study, risk rating of benzene was 5, showing very high rank of risk. Control approaches should be applied to these task groups. Effective engineering control, conduct air monitoring, conduct training for monitoring, and adopted respiratory protection program is recommended for control of very high rank (32).

Risk rating for H _2_ S, toluene, and xylene was 3, showing low rank of risk. Periodical assessment is recommended for control of low rank of risk every four years (32). Risk rating for pentane, hexane, heptane, octane, and nona was 1, showing negligible rank of risk. However, periodical assessment is recommended for control of negligible rank of risk every five years (32). The fundamental component of VOCs is benzene in petrochemical industries (17, 41).

According to our study, the cancer risk assessment of benzene exposures in the breathing zone was 8.78×10 ^−3^ , in other words, 8.7 cancer per 1000 i.e. higher than the acceptable criteria of 10 ^−6^ . Cancer risk for workers exposed to dangerous substances must not be more than 1.1 people per 100000 (23). Average cancer risk of benzene was higher than 10 ^−6^ in another study (41). Among VOCs compounds, benzene may result in carcinogenic risk (42). Therefore, long term exposure to VOCs (especially benzene) may result in a change in complete blood counts (CBC) (43, 44). In our study, non-cancer risk for BTX compounds was higher than the acceptable standard of one (adverse non-carcinogenic effects is concern). However, 3 risk factors can cause severity of cancer, cumulative risk, and aggregate exposure, such as occupational factors (industrial, farming, and laboratories), non-occupational factors (environmental, automobile, and mini-workshop), and individual factors (lifestyle, sex, age, BMI, and race).

## Conclusion

We did not consider cancer risk analysis for xylene, toluene, and H _2_ S in occupational environment because there was not appropriate method available to us. Although the rank of risk assessment in our study for major chemical substances was low, such periodical assessment is essential to apply control approaches. Risk assessment and cancer risk analysis methodologies before the operating phase of the industry can cause suggestions for changes in the industry system conditions and provide valuable information for planning, prioritize the ranking of hazard, and management programs.

## Ethical considerations

Ethical issues (Including plagiarism, informed consent, misconduct, data fabrication and/or falsification, double publication and/or submission, redundancy, etc.) have been completely observed by the authors.
